# Household saving during pregnancy and facility delivery in Zambia: a cross-sectional study

**DOI:** 10.1093/heapol/czz005

**Published:** 2019-02-14

**Authors:** Calvin Chiu, Nancy A Scott, Jeanette L Kaiser, Thandiwe Ngoma, Jody R Lori, Carol J Boyd, Peter C Rockers

**Affiliations:** 1 Innovations for Poverty Action Zambia, Plot 26, Mwambula Street, Jesmondine, Lusaka, Zambia; 2 School of Public Health, University of California, 2121 Berkeley Way, Berkeley, CA, USA; 3 Department of Global Health, Boston University School of Public Health, 801 Massachusetts Avenue, Boston, MA, USA; 5 Right to Care – Zambia, 11059 Off Brentwood Road, Longacres, Lusaka, Zambia; 6 University of Michigan School of Nursing, Center for Global Affairs & PAHO/WHO Collaborating Center, 426 N Ingalls St, Ann Arbor, MI, USA; 7 Alcohol, Smoking and Health, University of Michigan School of Nursing, Center for the Study of Drugs, 426 N Ingalls St, Ann Arbor, MI, USA

**Keywords:** Maternity services, costs, healthcare utilization, financial protection, Zambia

## Abstract

Financial barriers cause many women in low- and middle-income countries to deliver outside of a health facility, contributing to maternal and neonatal mortality. Savings accrued during pregnancy can increase access to safe delivery services. We investigated the relationship between household saving during pregnancy and facility delivery. A cross-section of 2381 women who delivered a child in the previous 12 months was sampled from 40 health facility catchment areas across eight districts in three provinces in Zambia in April and May of 2016. During a household survey, women reported on their perceptions of the adequacy of their household savings during their recent pregnancy. Households were categorized based on women’s responses as: did not save; saved but not enough; and saved enough. We estimated crude and adjusted associations between perceived adequacy of savings and facility delivery. We also explored associations between savings and expenditures on delivery. Overall, 51% of women surveyed reported that their household saved enough for delivery; 32% reported saving but not enough; and 17% did not save. Household wealth was positively associated with both categories of saving, while earlier attendance at antenatal care was positively associated with saving enough. Compared with women in households that did not save, those in households that saved but not enough (aOR 1.63; 95% CI: 1.17, 2.25) and saved enough (aOR 2.86; 95% CI: 2.05, 3.99) had significantly higher odds of facility delivery. Both categories of saving were significantly associated with higher overall expenditure on delivery, driven in large part by higher expenditures on baby clothes and transportation. Our findings suggest that interventions that encourage saving early in pregnancy may improve access to facility delivery services.

**Key Messages**

- Only half of women perceived that their household saved enough for their recent delivery.
- Perceived adequacy of savings was strongly associated with facility delivery.
- Saving enough was associated with reduced odds of reporting a problem related to delivery services.
- Saving was associated with increased expenditure on delivery.

## Introduction

Pregnant women in low- and middle-income countries often struggle to deliver at a health facility, contributing to high levels of maternal and neonatal mortality (Moyer and Mustafa, 2013; Tura *et al.*, 2013). There are ongoing efforts in many countries to increase access to delivery services by addressing supply side constraints. These include training more skilled birth attendants and refurbishing maternity wards and maternity waiting homes (USAID, 2016). However, pregnant women continue to face financial barriers to accessing safe delivery services, due to user fees (Dzakpasu *et al.*, 2014; Dodzo and Mhloyi, 2017), costs associated with transportation (Atuoye *et al.*, 2015; Kananura *et al.*, 2017), required delivery supplies and informal payments (Danilovich and Yessaliyeva, 2014; Dodzo and Mhloyi, 2017). Recent evidence suggests that delivering at a facility can cost as much as five times more than delivering at home (Skordis-Worrall *et al.*, 2011). These costs can produce a financial strain that can have long-term negative impacts on financial health post-partum (Hoque *et al.*, 2012; Dalinjong *et al.*, 2017) and disproportionately affects lower-income households (Ansong, 2015; Agunwa *et al.*, 2017; Benova *et al.*, 2017).

In many cases, savings must be built up during pregnancy to afford the costs associated with facility delivery. However, recent evidence suggests that many households struggle to save adequately for delivery. A recent study conducted among peri-urban households in Ghana and Nigeria found that less than one-third of households with a pregnant woman managed to save at all for their delivery (Jennings *et al.*, 2017). Although maternity care was nominally ‘free’ in Bangladesh, inadequate savings led many households to compensate by borrowing from a relative or money lender to pay for a range of hidden costs (Nahar and Costello, 1998). While recent attention has been given to the role of savings as a component of birth preparedness in Nigeria (Iliyasu *et al.*, 2010) and Uganda (Timsa *et al.*, 2015), there is limited evidence of the association between household savings and facility delivery (Moran *et al.*, 2006; Jennings *et al.*, 2017). While Cohen *et al*. (2017) found that pregnant women in Kenya intended to use higher-quality facilities when provided with financial assistance, there is also limited evidence showing improved access to higher-quality facilities due to increased savings.

Zambia is a lower-middle income country with 58% of the population living below the international poverty line of US$1.90 per day (World Bank, 2018). The overall fertility rate is 5.3 births per woman, with a substantially higher rate in rural areas (6.6) as compared with urban areas (3.7) [Central Statistical Office (CSO) *et al.*, 2014]. Two-thirds of births overall and 56% of births in rural areas occur at a health facility [Central Statistical Office (CSO) *et al.*, 2014]. The maternal mortality ratio in Zambia is high at 224 deaths per 100 000 live births (WHO *et al.*, 2015). User fees for primary health services including facility delivery were formally abolished in rural areas in 2006. However, there is mixed evidence regarding the resulting impact of user fee removal on healthcare utilization (Hangoma *et al.*, 2018; Lépine *et al.*, 2018). The costs of delivering at a health facility in Zambia go beyond user fees (Sialubanje *et al.*, 2014, 2015; Chama-Chiliba and Koch, 2016). In addition to paying for transportation to and from the facility, pregnant women are often told during antenatal care visits that they are required to purchase and bring supplies (e.g. cotton wool, surgical gloves and disinfectant) to the facility for delivery (Scott *et al.* 2018a). Some women choose to stay at maternity waiting homes prior to delivery, which are meant to have no formal fees in Zambia but in some cases have costs, including for meals (Lori *et al.* 2016; Scott *et al.* 2018b). Finally, women in Zambia have reported embarrassment and public shame as a result of delivering at a facility without having new clothing for their baby, and as a result many women purchase new clothes which can be quite expensive (Scott *et al.* 2018b). In recent years, the Saving Mothers Giving Life (SMGL) programme has been rolled out throughout much of Zambia, including in the study area (Kruk *et al.*, 2016). A multi-donor programme aimed at reducing maternal mortality, SMGL involves activities to increase demand for and access to facility delivery, including birth preparedness activities with some savings-related content.

In this article, we explore socio-demographic factors associated with saving for delivery and assess the relationship between savings accrued during pregnancy and facility delivery. We also investigate the association between savings and expenditures on delivery. Our findings have implications for policy discussions around safe delivery in low- and middle-income countries like Zambia.

## Materials and methods

### Study population

Data were collected during baseline of an ongoing cluster randomized controlled trial evaluating the impact of a maternity waiting home intervention in April and May of 2016 [clinicaltrials.gov identifier: (NCT02620436)]. The trial covers 40 health facility catchment areas in Choma, Kalomo and Pemba districts in Southern Province, Nyimba and Lundazi districts in Eastern Province, and Mansa and Chembe districts in Luapula Province and has been described extensively elsewhere (Lori *et al.*, 2016; Scott *et al.*, 2018a). Households were identified using a multi-stage random sampling procedure. In the first stage of sampling, every village within the study facility catchment areas were visited and GPS co-ordinates were taken. Co-ordinates were then used to determine the distance of each village center to their designated health facility by travel distance along the most direct route, and only villages at least 10 km (rounding up from 9.5 km) from their designated health facility were considered in subsequent stages of sampling. Ten villages were randomly selected from each catchment area with probability proportional to population size. In the second stage of sampling, an exhaustive list of households that had delivered in the previous 12 months was created with input from the facility and traditional leadership, and the households were randomly ordered. Each household was then visited in that random order and confirmed for eligibility. The process continued down the list until approximately six eligible households were enrolled in each village. To be eligible, women had to have delivered a child during the previous 12 months and be at least 15 years old. If a household had more than one eligible participant, one respondent was selected at random by the electronic data capture system. The target sample size was 2400. Ethical approvals were obtained from the [Boston University] Institutional Review Board (Protocol number [H-34526]) and ERES Converge in Zambia (Protocol number [2015-Dec-012]).

### Variables

Household savings was measured using a nested pair of closed-ended questions. Women were first asked whether they had ‘money set aside in preparation for [their] last delivery’. Those who answered ‘yes’ were then asked whether they thought they had ‘saved enough money’. For the analysis, responses were coded as perceived adequacy of savings with three categories: did not save; saved but not ‘enough’; and saved ‘enough’. These questions captured respondents’ perceptions and were not corroborated with objective estimates of savings values. Questions on savings were designed to elicit information on household savings, not only a women’s personal savings, and additional data were collected on where savings were stored and who else contributed. Information on when during pregnancy saving started, measured by gestational age in months, was also collected. Finally, women reported whether they had ever saved at a bank, and rated the importance of saving for delivery using a likert scale from not important to very important.

The primary outcome of interest was delivery at a health facility. We recorded the location of each woman’s last delivery, including the facility where she delivered if she delivered at a health facility. Analysis focused on the dichotomous outcome of whether delivery occurred at a health facility, regardless of type or location. Perceived quality of delivery services is also an outcome of interest, and was measured among women who delivered at a health facility. Women were asked whether or not they experienced problems with the quality of the following: technical quality of medical care received, respect shown by healthcare workers, privacy during delivery, and cleanliness of the healthcare facility. These data were analysed as a dichotomous variable indicating whether or not pregnant women reported at least one problem with their delivery facility. We also explore the relationship between perceived adequacy of savings and expenditures on delivery. Data on several expenditure categories related to delivery were collected in the local currency (Zambian Kwacha, ZMW): delivery supplies, baby clothes, transportation, stay at a maternity waiting home, provider/health center fees, informal payments, tips, in-kind resources, drugs, diagnostic tests and other fees.

We collected demographic characteristics at the individual and household levels to investigate associations with savings behaviour. This included age, years of education, distance from designated clinic (in kilometers), marital status, number of household members, parity, gravida, HIV status and household wealth. Marital status and HIV status were coded as dichotomous indicators. An indicator of household wealth was constructed with principle component analysis of asset information. All other demographic variables were included as continuous variables in the analysis.

We collected data on the number of antenatal care visits each woman attended during her pregnancy, and when she attended her first session measured by gestational age in months. Lastly, we asked each woman where she had intended to deliver, and who the primary decision maker was for the location of delivery. This was coded as a dichotomous variable indicating whether or not the woman herself was the primary decision maker.

### Analysis

First, we calculated descriptive statistics, stratifying by perceived adequacy of savings during pregnancy. We conducted a set of *t*-tests comparing participant characteristics across categories of perceived adequacy of savings. Next, we fit a series of logistic regression models to estimate crude and adjusted associations between perceived adequacy of savings and facility delivery. Among those who delivered at a health facility, we repeated the analysis to estimate the relationship between savings and perceived quality of delivery services. Finally, we summarized expenditures on delivery and conducted a set of *t*-tests comparing expenditures across savings categories. All models were fit using Stata version 14 (College Station, TX, USA: StataCorp LP). All standard errors were clustered at the village level to account for the study design.

## Results

Table 1 presents descriptive statistics for the study population (*n* = 2381). Just over half (51%) of women reported that their household saved enough for their last delivery; 32% reported that their household saved but not enough; and 17% reported that their household did not save at all. Nearly all women reported that saving for delivery was important (Supplementary Table A1). Among those who reported saving anything for delivery, the vast majority reported storing their savings at home (91%) and that others contributed towards their savings (95%). Other contributors included the husband (90%) and parents and grandparents (17%) (Supplementary Table A2). Households started saving on average around their fifth month of pregnancy. While 99% of women reported intending to deliver at a health facility, 81% did so. Among those who delivered at a health facility, 29% reported at least one problem with the quality of the delivery services. Women attended an average of 3.7 antenatal care visits, the first of which was reported to occur approximately at 4 months gestation on average.

**Table 1:** Characteristics of study participants

	Mean	(SD)	*N*
Demographics			
Age^a^ (years)	26.11	(6.97)	2372
Education (years completed)	6.25	(2.31)	2012
Distance from nearest clinic (km)^b^	15.45	(9.34)	2375
Married, *n* (%)	2092	88%	2376
Number of household members	6.99	(3.58)	2381
Number of live births	3.59	(2.35)	2379
Primagravida*, n* (%)	508	21%	2379
HIV positive, *n* (%)	50	2%	2246
Household assets			
Non-improved water source,^c^*n* (%)	1336	56%	2379
Non-improved toilet,^d^*n* (%)	2140	90%	2380
No electricity, *n* (%)	2368	99%	2381
Earth or sand floors, *n* (%)	2094	88%	2378
Charcoal or wood cooking fuel, *n* (%)	2368	99%	2379
Savings			
Perceived adequacy of savings, *n* (%)			
Did not save	412	17%	2361
Saved but not enough	755	32%	2361
Saved enough	1194	51%	2361
Ever saved at a bank, *n* (%)	106	4%	2373
Amongst those who reported saving			
Savings were stored at home, *n* (%)	1780	91%	1954
Months pregnant when started saving	4.95	1.81	1954
Others contributed towards delivery savings, *n* (%)	1340	95%	1414
Health seeking			
Intended to deliver at a healthcare facility, *n* (%)	2355	99%	2375
Delivered at a healthcare facility, *n* (%)	1934	81%	2376
Self as primary decision maker for site of delivery, *n* (%)	860	36%	2381
Months pregnant when attended first ANC visit	4.20	1.46	2356
Number of ANC visits	3.65	1.06	2374
Perceived quality of delivery services			
Problem with technical quality of medical care received, *n* (%)	337	18%	1919
Problem with respect shown by healthcare workers, *n* (%)	304	16%	1919
Problem with privacy during delivery, *n* (%)	195	10%	1924
Problem with cleanliness of healthcare facility, *n* (%)	183	10%	1925
Problem with at least one of the above, *n* (%)	555	29%	1926

^a^Age ranged from 15- to 49-years old.

^b^Distance from the nearest clinic ranged from 8 to 89 km.

^c^Non-improved water source: unprotected dug well, unprotected spring, tanker truck, cart with small tank, surface water.

^d^Non-improved toilet: pit latrine without slab/open pit, bucket toilet, hanging toilet/latrine, no facility/bush/field.

Associations between demographic characteristics and perceived adequacy of household savings are presented in Table 2. Household wealth and being married were positively associated with both categories of savings. Earlier attendance at first antenatal care visit was positively associated with saving enough. Women who reported being HIV positive were significantly less likely to report that their household saved enough.

**Table 2:** Perceived adequacy of savings and demographic characteristics

	Did not save Mean (SE) (1)	Saved but not enough Mean (SE) (2)	Saved enough Mean (SE) (3)	(2)–(1)		(3)–(1)		(3)–(2)	
				Mean (SE)	*P*-value^1^	Mean (SE)	*P*-value^1^	Mean (SE)	*P*-value^1^
Age	25.76 (0.37)	26.48 (0.26)	26.01 (0.21)	0.72 (0.49)	0.14	0.27 (0.42)	0.52	−0.45 (0.36)	0.21
Years of education	6.12 (0.13)	6.25 (0.10)	6.30 (0.09)	0.13 (0.19)	0.47	0.18 (0.19)	0.36	0.04 (0.16)	0.80
Distance from nearest clinic (km)	15.84 (0.59)	15.25 (0.43)	15.48 (0.54)	−0.59 (1.14)	0.61	−0.36 (1.51)	0.81	0.23 (1.21)	0.85
Probability of being married	0.82 (0.02)	0.88 (0.01)	0.90 (0.01)	0.06 (0.02)	0.01	0.08 (0.02)	0.00	0.02 (0.01)	0.17
Number of household members	6.92 (0.20)	7.11 (0.13)	6.90 (0.13)	0.18 (0.22)	0.42	−0.03 (0.26)	0.91	−0.21 (0.21)	0.31
Parity	3.49 (0.12)	3.78 (0.09)	3.51 (0.07)	0.29 (0.16)	0.07	0.03 (0.13)	0.81	−0.26 (0.13)	0.04
Primagravida	0.22 (0.02)	0.19 (0.02)	0.22 (0.01)	−0.03 (0.03)	0.38	0.00 (0.02)	0.99	0.03 (0.02)	0.23
Wealth z-score	−0.32 (0.05	−0.01 (0.04)	0.12 (0.04)	0.30 (0.07)	0.00	0.43 (0.08)	0.00	0.13 (0.07)	0.07
Probability of HIV positive	0.03 (0.01)	0.03 (0.01)	0.01 (0.00)	0.00 (0.01)	0.83	−0.02 (0.01)	0.06	−0.02 (0.01)	0.02
Self as primary decision maker for site of delivery	0.38 (0.03)	0.37 (0.02)	0.35 (0.02)	−0.01 (0.04)	0.75	−0.03 (0.03)	0.30	−0.02 (0.03)	0.44
Months pregnant at time of first ANC visit	4.41 (0.08)	4.30 (0.06)	4.06 (0.05)	−0.11 (0.12)	0.37	−0.34 (0.11)	0.00	−0.23 (0.09)	0.01
Number of ANC visits attended	3.48 (0.06)	3.66 (0.04)	3.70 (0.03	0.18 (0.07)	0.02	0.22 (0.07)	0.00	0.05 (0.06)	0.42
Clusters	425	425	425						
Observations	412	755	1194						

^a^Based on *t*-tests of mean difference between groups. Standard errors are clustered at the village level.

Compared with women in households that did not save, those in households that saved but not enough had significantly higher odds of delivering at a health facility (aOR 1.63; 95% CI: 1.17, 2.25; Table 3). Those in households that saved enough had the highest odds of delivering at a facility (aOR 2.86; 95% CI: 2.05, 3.99). Among women who delivered at a facility, those in households that saved enough were significantly less likely to report any problem with service quality (aOR 0.65; 95% CI: 0.46, 0.92). This is driven primarily by problems with respect shown by healthcare workers (Supplementary Table A3).

**Table 3:** Perceived adequacy of savings and facility delivery

	% of pregnant women who			Intended to deliver at a facility		Delivered at a facility		Reported any problem with delivery facility	
	Intended to deliver at facility	Delivered at a facility	Reported any problem with delivery facility						
				Crude OR (95% CI)	Adjusted^a^ OR (95% CI)	Crude OR (95% CI)	Adjusted^a^ OR (95% CI)	Crude OR (95% CI)	Adjusted^a^ OR (95% CI)
Level of savings									
Did not save	98.3	68.9	31.8	Ref	ref	Ref	ref	ref	ref
Saved but not enough	99.2	79.3	34.8	2.15 (0.72, 6.43)	2.27 (0.56, 9.22)	1.73*** (1.32, 2.27)	1.63** (1.17, 2.25)	1.14 (0.84, 1.56)	1.06 (0.74, 1.52)
Saved enough	99.2	87.0	24.6	2.27 (0.90, 5.75)	1.86 (0.56, 6.04)	3.01*** (2.30, 3.94)	2.86*** (2.05, 3.99)	0.70* (0.52, 0.93)	0.65* (0.46, 0.92)
Clusters	–	–	–	425	392	425	394	414	384
Observations	2 353	1934	1914	2356	1836	2357	1877	1911	1549

*P < 0.05, **P < 0.01. Standard errors are clustered at the village level.

^a^Controlling for: age, years of education, distance from nearest clinic, marital status, household members, parity, primagravida, household wealth, HIV status, delivery location decision maker status, months pregnant at time of first antenatal care visit and number antenatal care visits.

Associations between perceived adequacy of savings and household expenditures on delivery are presented in Table 4. Households that did not save spent an average of ZMW 189 on delivery. Compared with those households, those that saved but not enough spent an additional ZMW 113 on delivery (*P* < 0.01), while those that saved enough spent an additional ZMW 135 (*P* < 0.01). Additional expenditure among households that saved was driven primarily by spending on baby clothes and transportation. There were no significant differences in expenditures between households that saved but not enough and those that saved enough.

**Table 4:** Perceived adequacy of savings and expenditures on delivery

Expenditure category	Did not save Mean (SE) (1)	Saved but not enough Mean (SE) (2)	Saved enough Mean (SE) (3)	(2)–(1)		(3)–(1)		(3)–(2)	
				Mean (SE)	*P*-value^a^	Mean (SE)	*P*-value^a^	Mean (SE)	*P*-value^a^
Supplies	34.81 (3.43)	36.95 (2.54)	44.36 (2.89)	2.14 (4.27)	0.62	9.56 (6.60)	0.15	7.41 (4.62)	0.11
Baby clothes	144.74 (20.86)	233.23 (15.33)	242.02 (10.52)	88.49 (25.89)	0.00	97.31 (13.01)	0.00	8.82 (16.82)	0.60
Transportation	11.44 (2.84)	30.10 (2.10)	36.97 (2.98)	18.65 (3.53)	0.00	25.56 (5.42)	0.00	6.91 (4.72)	0.14
Stay at a maternity waiting home	0.47 (0.59)	1.62 (0.44)	2.78 (0.51)	1.15 (0.73)	0.12	2.32 (1.16)	0.05	1.17 (0.82)	0.15
Provider/health center fees	0.44 (0.39)	0.70 (0.29)	0.84 (0.23)	0.26 (0.49)	0.60	0.40 (0.44)	0.36	0.15 (0.37)	0.69
Informal payments	0.45 (0.65)	1.08 (0.48)	0.30 (0.30)	0.63 (0.80)	0.43	−0.15 (0.18)	0.40	−0.79 (0.48)	0.10
Tips	0.01 (0.01)	0.01 (0.01)	0.07 (0.02)	−0.00 (0.01)	0.92	0.05 (0.05)	0.31	0.06 (0.04)	0.16
In-kind resources	0.05 (0.17)	0.18 (0.12)	0.09 (0.06)	0.13 (0.21)	0.52	0.04 (0.07)	0.56	−0.09 (0.09)	0.34
Drugs	0.02 (0.05)	0.02 (0.04)	0.31 (0.15)	−0.00 (0.06)	0.94	0.28 (0.33)	0.39	0.29 (0.24)	0.24
Diagnostic tests	0.00 (0.06)	0.05 (0.05)	0.72 (0.53)	0.04 (0.08)	0.56	0.72 (1.15)	0.53	0.67 (0.85)	0.43
Other fees	0.23 (0.61)	2.34 (0.45)	1.12 (0.39)	2.11 (0.76)	0.01	0.90 (0.63)	0.16	−1.22 (0.64)	0.06
Total	188.84 (21.42)	301.97 (15.82)	324.20 (11.56)	113.12 (26.63)	0.00	135.42 (18.02)	0.00	22.29 (18.47)	0.23
Clusters	425	425	425						
Observations	412	755	1194						

^a^Based on *t*-tests of mean difference between groups. Standard errors are clustered at the village level.

## Discussion

We investigated the relationship between household saving during pregnancy and facility delivery among a sample of women in rural Zambia. Our analysis yielded two main findings. First, only half of women in this population perceived that their household saved enough for their delivery. Second, perceived adequacy of savings accrued during pregnancy was strongly associated with facility delivery. Our findings are consistent with previous research that shows that financial barriers are key contributors to low rates of facility delivery in low- and middle-income countries (Mrisho *et al.*, 2007; Gabrysch and Campbell 2009), including in settings like Zambia where user fees have been abolished (Kruk *et al.*, 2008; Perkins *et al.*, 2009). Our findings have implications for global efforts to achieve Universal Health Coverage, which has a financial protection dimension and a service coverage dimension (Saksena *et al.*, 2014). Women in households that did not save for delivery reported an average expenditure of 189 ZMW (∼US$20), a significant outlay for the average household in rural Zambia, where per capita income is around US250 per year (Central Statistical Office Zambia, 2015). A large proportion of reported expenditure was on new clothing for the baby, which previous research suggests is very important to new mothers in rural Zambia (Scott *et al*., 2018b). In addition to inhibiting a woman’s ability to access facility-based safe delivery services, insufficient savings during pregnancy may also have long-lasting negative impacts on household financial health.

There is significant room for improvement in saving behaviour; only 51% of women in our study reported that their household saved enough. This is particularly concerning given the near universal (99%) agreement that saving money is important for delivery. Study households were located at least 10 km from the nearest health facility and were likely poorer than the average Zambian household (Gabrysch *et al.*, 2011), and we expect that most if not all would have benefited from saving during pregnancy. On average, households started saving around 5 months into pregnancy, which leaves limited time to achieve saving goals, especially if the woman experiences birth complications and is required to deliver ahead of her due date. We found that greater household wealth and being married were associated with saving, likely a result of access to greater financial resources. Saving was associated with a modest increase in spending on supplies and transportation, and a large increase in spending on baby clothes. This may suggest that savings can spare women embarrassment from not being able to properly clothe their baby in a public setting, a source of social shame in Zambia that women have previously indicated was a reason for delivering at home (Scott *et al.*, 2018b). Increases in expenditures associated with saving were similar whether the respondent perceived that the household had saved enough or not. This underscores the subjective nature of women’s perceptions on what constitutes adequate savings for delivery. There may be opportunities to integrate savings-focused interventions into existing programmes that aim to increase facility delivery rates in Zambia, e.g. SMGL. New research on potential interventions focused on increasing savings during pregnancy may be warranted.

Saving enough was associated with reduced odds of reporting a problem related to delivery services. There are a few plausible explanations for this relationship. First, increased savings may provide women with the financial means to bypass their nearest health facility and access a more preferred and higher-quality facility, i.e. more distant. Frequent bypassing of this type has been documented in nearby Tanzania (Kruk *et al.*, 2009). Second, recent research in Zambia has documented evidence that some women feel mistreated by health facility staff when they arrive for delivery without the supplies that they have been told to bring during antenatal care (Sialubanje *et al.*, 2015). We found a particularly strong negative association between saving enough and reporting a problem related to feeling respected by facility staff, which suggests that this second explanation may be particularly important.

This analysis has several important limitations. First, our main measure of savings relied on women’s perceptions, and we are not able to corroborate those perceptions with data on actual savings values. Ideally, we would have liked to collect a more robust measure of savings to compare with expenditure data. However, women’s perceptions of their own birth preparedness likely plays an important role in their decision on whether to deliver at a health facility, independent of how their perceptions compare with those of other women in their community. Second, the cross-sectional nature of our analysis precludes a strong causal interpretation of our results. This is true for the primary relationship of interest between household savings and facility delivery. We are also not able to establish causal directionality in the association between early first attendance at antenatal care and saving enough for delivery. Antenatal care sessions may have provided information about birth planning and encouraged pregnant women to save early for delivery to purchase the necessary supplies to deliver at a health facility. Alternatively, women who attend antenatal care sessions early may have been more committed to delivering at a facility or more able to save. Third, only women in villages at least 10 km from their health facility were included in the study because the parent trial was focused on this population. As a result, our findings may not be generalizable beyond households in remote communities. These households are on average poorer and less educated than less-remote households (Gabrysch *et al.*, 2011), and may face greater difficulty saving for delivery and greater financial barriers to accessing facilities. Fourth, we are not able to determine the extent to which women controlled the savings that they reported, as opposed to their husband or another member of their household controlling it. Further research is needed to shed light on the intra-household dynamics of saving decisions during pregnancy. Lastly, our measure of service quality relies on perceived problems by pregnant women and we do not have complementary objective data on quality such as the BEmONC signal functions (Otolorin *et al.*, 2015). However, perceived problems such as respect shown by healthcare workers reflect women’s subjective preferences and may more directly affect the decision-making process on where to deliver than objective indicators on which women may have limited information.

## Conclusions

Our findings demonstrate a strong association between perceived adequacy of savings and delivery at a health facility in rural Zambia. Efforts that focus on overcoming financial barriers through savings may complement existing demand generation strategies. However, the poorest households may be particularly disadvantaged when it comes to saving, and savings interventions should perhaps be coupled with other efforts to reduce financial barriers that explicitly target the poorest, e.g. providing supplies at facilities free of charge. Future research should focus on exploring interventions that encourage saving early in pregnancy to improve access to safe delivery services.

## Funding

This work was supported by, developed, and is being implemented in collaboration with *MSD for Mothers*, MSD’s 10-year, $500 million initiative to help create a world where no woman dies giving life. *MSD for Mothers* is an initiative of Merck & Co., Inc., Kenilworth, NJ, USA [MRK 1846–06500.COL]. This work was additionally supported in part by the Bill & Melinda Gates Foundation [OPP1130329] and The ELMA Foundation [ELMA-15-F0017]. The funders had no role in study design, data collection and analysis, decision to publish or preparation of the manuscript. The content is solely the responsibility of the authors and does not reflect positions or policies of MSD, the Bill & Melinda Gates Foundation or The ELMA Foundation.

## Supplementary Material

Supplementary Appendix
