# Supplementary material for: Household saving during pregnancy and facility delivery in Zambia: a cross-sectional study
Source: Health Policy Plan. 2019 Feb 14;34(2):102–9. doi: 10.1093/heapol/czz005 (PMC6481286; doi:10.1093/heapol/czz005)
Supplement: Supplementary Appendix [file czz005_supplementary_appendix.docx]

## **Appendix**

## **Table A1.** Beliefs regarding the importance of saving money for delivery

| **How important is it to save for delivery** | **N** | **%** |
| --- | --- | --- |
| Not important | 16 | 1% |
| Slightly important | 11 | 0% |
| Moderately important | 14 | 1% |
| Important | 433 | 18% |
| Very important | 1,900 | 80% |
| **Total** | 2,374 | 100% |

## **Table A2.** Contributors to delivery savings

|  | **N** | **%** |
| --- | --- | --- |
| Husband/partner | 1,358 | 90% |
| Children | 5 | 0% |
| Parent/grandparent | 247 | 17% |
| Other family member | 72 | 5% |
| Friend | 10 | 11% |
| Auntie | 6 | 0% |
| Other | 4 | 0% |
| No one | 74 | 5% |

**Table A3.** Perceived adequacy of savings and reporting of problems with delivery facility

|  | **Problem with technical quality of medical care** | | **Problem with respect shown by healthcare workers** | | **Problem with privacy during delivery** | | **Problem with cleanliness of healthcare facility** | |
| --- | --- | --- | --- | --- | --- | --- | --- | --- |
|  | **Crude OR (95% CI)** | **Adjusted OR (95% CI)^1^** | **Crude OR (95% CI)** | **Adjusted OR (95% CI)^1^** | **Crude OR (95% CI)** | **Adjusted OR (95% CI)^1^** | **Crude OR (95% CI)** | **Adjusted OR (95% CI)^1^** |
| **Perceived adequacy of savings** |  |  |  |  |  |  |  |  |
| Did not save | ref | Ref | ref | ref | ref | ref | Ref | ref |
| Saved but not enough | 1.13  (0.78, 0.62) | 1.04  (0.68, 1.59) | 1.03  (0.74, 1.44) | 0.96  (0.65, 1.43) | 0.84  (0.54, 1.32) | 0.83  (0.46, 1.48) | 1.23  (0.77, 1.94) | 1.11  (0.66, 1.87) |
| Saved enough | 0.72  (0.51, 1.02) | 0.62*  (0.41, 0.94) | 0.58**  (0.41, 0.82) | 0.54**  (0.35, 0.84) | 0.63*  (0.40, 0.98) | 0.71  (0.40, 1.27) | 0.87  (0.57, 1.35) | 0.93  (0.55, 1.56) |
| Clusters | 414 | 384 | 414 | 384 | 413 | 384 | 414 | 384 |
| Observations | 1,904 | 1,543 | 1,904 | 1,545 | 1,909 | 1,549 | 1,910 | 1,548 |

*Notes:* * p<0.05, **p<0.01. Standard errors are clustered at the village level

^1^Controlling for: age, years of education, distance from nearest clinic, marital status, household members, parity, primagravida, household wealth, HIV status, delivery location decision maker status, months pregnant at time of first antenatal care visit, and number antenatal care visits.
